# The influence of maternal agency on severe child undernutrition in conflict-ridden Nigeria: Modeling heterogeneous treatment effects with machine learning

**DOI:** 10.1371/journal.pone.0208937

**Published:** 2019-01-09

**Authors:** Nadine Kraamwinkel, Hans Ekbrand, Stefania Davia, Adel Daoud

**Affiliations:** 1 Department of Sociology and Work Science, University of Gothenburg, Gothenburg, Sweden; 2 Harvard Center for Population and Development Studies, T.H. Chan School of Public Health, Harvard University, Boston, Massachusetts, United States of America; 3 The Alan Turing Institute, London, United Kingdom; The University of Warwick, UNITED KINGDOM

## Abstract

Nigeria is one of the fastest growing African economies, yet struggles with armed conflict, poverty, and morbidity. An area of high concern is how this situation affects vulnerable families and their children. A key pathway in improving the situation for children in times of conflict is to reinforce maternal agency, for instance, through education. However, the state of the art of research lacks a clear understanding of how many years of education is needed before children benefit. Due to mother’s differing social context and ability, the effect of maternal education varies. We study the heterogeneous treatment effects of maternal agency, here operationalized as length of education, on severe child undernutrition in the context of armed conflict. We deploy a repeated cross-sectional study design, using the Nigeria 2008 and 2013 Demographic and Health Survey (DHS). The sample covers 25,917 children and their respective mothers. A key methodological challenge is to estimate this heterogeneity inductively. The causal inference literature proposes a machine learning approach, Bayesian Additive Regression Trees (BART), as a promising avenue to overcome this challenge. Based on BART-estimation of the Conditional Average Treatment Effect (CATE) this study confirms earlier findings in that maternal education decreases severe child undernutrition, but only when mothers acquire an education that lasts more than the country’s compulsory 9 years; that is 10 years of education and higher. This protective effect remains even during the exposure of armed conflict.

## Introduction

Nigeria currently constitutes the largest economy in Africa with abundant human capital and economic potential. Yet, its under-five child mortality level is among the highest in Sub-Saharan Africa [1]. Although child mortality has decreased from 213 per 1000 in 1990 to 128 per 1000 in 2013, the Sustainable Development Goals (SDG) target of 25 per 1000 live births remains out of reach for Nigeria [2]. Large geographical disparities remain with mortality and poverty being more pronounced in rural sites and in Northern Nigeria more than Southern [2, 3].

Children’s living conditions tends to worsen under the influence of adverse events. Typically, their access to food can decrease, leading to rapid and adverse consequences on their health. Nigeria faces extreme weather conditions, political instability, and recurrent armed conflict [4]. This year, Nigeria is entering its ninth year of ongoing crisis related to Boko Haram (*books are forbidden*) and The Civilian Joint Task Force violence. This conflict caused an estimated 3.9 million people in Nigeria to become food insecure or deprived and more than 400,000 children to suffer from severe and acute undernutrition [5]. A recent study examining the changing undernutrition profile in Nigeria showed that the prevalence of multiple anthropometric failure increased from 23 percent to 28 percent between 2008 and 2013 [6], which may be partly due to the increase in conflict. Nigeria’s context highlights the complexity involved in addressing child health in fragile settings.

An integral aspect of securing the child’s health and safety are the parents. Mothers specifically have a crucial role in the child’s living conditions. Past studies suggest that women’s agency can play an important role for strategic investments in the child’s nutrition, education and health [7–11]. Maternal agency can be defined as the ability of a mother to use, direct, and control resources, to make wise choices, and to formulate and put ideas that prioritize personal opinion, needs, and well-being into practice [8]. With this definition in mind and the knowledge that maternal education is strongly associated with better health, nutrition, and well-being outcomes for young children [10, 12], we assert that for the purpose of this paper, maternal education as an applicable proxy for maternal agency.

Investments in education for women have proved beneficial in several ways. Opportunities and employability increase with years of school and lead to improvements in women’s socioeconomic standing. In regard to child care, education improves the mother’s knowledge about child health, prevention, and treatment of disease [13]. Educated women are more likely to be aware of the importance of vaccinating their children, feeding the child the right foods, and taking early action when the child is sick [12], thereby giving their child the best available opportunity to grow and thrive.

Although the literature indicates that mother’s education has a positive impact on child nutrition [11], there is no consensus on its pathways and little research has been done on how many years of schooling are necessary for mothers to sustain their children’s health [14]. A study by Makoka and Masibo [10] utilized Demographic and Health Survey (DHS) data from Malawi, Tanzania, and Zimbabwe to analyze the influence of various levels of maternal education on child nutrition outcomes (i.e. stunting, underweight, and waisting). They found that higher levels of maternal education reduced the odds of stunting, but only when mothers had obtained at least ten years of schooling, whereas the threshold was lower for the risk on wasting and underweight [10]. These findings indicate treatment heterogeneity of maternal education on child nutritional status.

The aim of this paper is to analyze treatment heterogeneity of maternal agency (i.e. education) on severe child undernutrition, and how this effect plays out in the context of armed conflict. We apply Bayesian Additive Regression Trees (BART) to disentangle this effect, and utilize observational data from DHS for Nigera 2008 and 2013.

## Methods

The analysis is based on four data sources: micro level data provided by the Demographic and Health Survey (DHS), the International Wealth Index provided by Global Data Lab [15], coordinate-based data on armed conflicts from the Uppsala Conflict Data Program [16], and coordinate-based data on environmental conditions from The African Flood and Drought Monitor (AFDM) [17]. The Global Living Conditions (GLC) R package [18] was used to harmonize DHS data and merge all data sources into one dataset. Descriptions of the data and the measures calculated from each data source are described first, followed by an explanation of the approach used to estimate the treatment effects.

### Child, maternal, and household data

We use child, maternal and household data from the Demographic and Health Survey (DHS). The DHS program aims to capture a representative sample of the population and provides health and population information, including nutritional status of young children and education of the mother for a large number of countries. Important for the purpose of this study is that the data also includes the geographical location of the respondent, which is essential to estimate the child’s exposure to conflict. For anonymity reasons, the coordinates provided by DHS are randomly displaced, however the displacement is always less than 20 km. The DHS is on average conducted every five years. Nigeria has five repeated cross-section surveys. We use two of the most recent rounds, 2008 and 2013, which represents the best current available data with coordinates to measure the influence of Nigeria’s conflict on the relationship between maternal agency (i.e. education) and child nutritional status. An overview of all the household, mother and child characteristics can be retrieved from S1 Appendix.

#### Severe child undernutrition

While the conventional indicators of stunting, wasting, and underweight provide context to child undernutrition, the Composite Index of Anthropometric Failure (CIAF) provides a more useful picture of undernutrition in children to investigate the function of food deprivation and disease. In this study, a means of identifying all undernourished children is required, thus the solution is using CIAF. Namely, when anthropometric indicators are used as stand-alone proxies, they may cause over- and underestimation of child nutritional status [19]. Theoretically put, it is the severe deprivation of food that likely effectuates serious adverse health and development outcomes for children [20]. Therefore, we apply the methodology from the Bristol approach [20], which is a versatile method for measuring child poverty, and is based on the internationally agreed definition (adopted at the 1995 World Summit on Social Development in Copenhagen) of absolute child poverty. The approach includes a method for estimating child food deprivation, and we applied this to capture the response variable, severe child undernutrition.

The weight, length, age and sex of each child are used to calculate whether the child suffers from severe undernutrition. With these variables, the nutritional indicators (I) weight for age and sex, (II) height for age and sex, and (III) weight for height, age, and sex are calculated for each child, using the method as laid out by the World Health Organization (WHO) Child Growth Standards [21]. Since we are interested in severe undernutrition we used the GLC package [18] to transform these nutritional indicators into a single binary measurement y_i_ ∈ {1, 0}. For a child to be classified as severely food deprived according to the Bristol method (i.e. severe undernutrition) it needs to fall more than -3 standard deviations below the median of the international reference (i.e. severe anthropometric failure), on any of the above nutritional indicators.

#### Maternal agency

The proxy for maternal agency, the mother’s educational level, is obtained by taking the DHS variable ‘education in years’, and constructing a categorical variable for maternal education, with values “no education” (0 years), “up to and including primary education” (1-6 years), “lower secondary education” (7—<9 years), “completed lower secondary education” (9 years, Nigeria’s educational law), “up to and including higher secondary education” (10-12 years), and “college and higher” (more than 12 years). This categorical variable is used as primary explanatory variable in our analysis of the effect of maternal agency on the child’s probability for severe undernutrition, and we model this to be a treatment. The cut-offs were established taking in consideration Nigeria’s nine year compulsory education system and to obtain cleaner comparison of the effect of maternal education and, as mentioned previously, earlier work demonstrated that more than 10 years of schooling are necessary to reduce stunting in children [10].

#### Mothers characteristics

Since maternal agency and child undernutrition might differ from mother to mother, characteristics including mother’s age, religion, native language, and breastfeeding were also included as covariates. With the variable native language we assume that it can capture the ethnicity of the mother, as Nigerian languages can be very cultural specific. Breastfeeding secures the infants nutritional status and prevents the onset of morbidity and mortality in infancy and early childhood caused by illnesses such as diarrhea [22].

#### Household characteristics

With respect to household characteristics, we include the DHS variables geographical location (rural/urban), International Wealth Index (IWI), and electricity status. Poor access to water, sanitation, and hygiene (WASH) can increase the child’s risk on severe undernutrition through morbidity [23]. A more recent study found that good access to toilets, but not water, yielded less risk on stunting in children, and this was found for one year old children up to five and eight years old [24]. For these reasons we include from the Bristol method the indicator severe sanitation deprivation. Severe sanitation deprivation {1, 0} is assigned to children who have ‘no access to a toilet of any kind in the vicinity of their dwelling, that is, no private or communal toilets or latrines’ [20]. Furthermore, we include the International Wealth Index provided by Global Data Lab [15] as the DHS provided wealth index is not comparable across surveys. The international wealth index is derived from Principal Component Analysis of survey responses to multiple questions regarding asset ownership, of which many are observable to the surveyor, and is able to capture the economic status of the household [15]. Similarly, electricity status and geographical location are indicators of (access to) resources. Furthermore, we include two variables that describe the geographical location of the household to which the child belongs: the administrative region and a variable with higher geographical resolution. The latter is generated by creating a voronoi partitioning of Nigeria on the basis of survey density whereby areas are merged until every area consists of a DHS survey from 2008 and 2013.

#### Child characteristics

Our analysis also includes child characteristics: age and sex of the child, the child’s relation to the head of the household. The child’s relationship with the household head can provide us with information on whether the observation is a son/daughter or adopted, among others. All of the aforementioned household, mother and child characteristics are utilized as control factors in our model.

### Conflict data

To develop the explanatory variable armed conflict intensity we use data from the Uppsala Conflict Data Program (UCDP)/PRIO Armed Conflict Dataset (version 5) [16]. This database is transparent in its construction and records all conflicts with a threshold of 25 battle-related deaths per year, such that it also includes minor conflicts. The definition of armed conflict is “a contested incompatibility that concerns government or territory or both where the use of armed force between two parties, of which at least one is the government of a state, results in at least 25 battle-related deaths” [16]. The intensity of armed conflict for a given area at a given point in time is not directly observable, instead it must be inferred from data.

**Fig 1:**
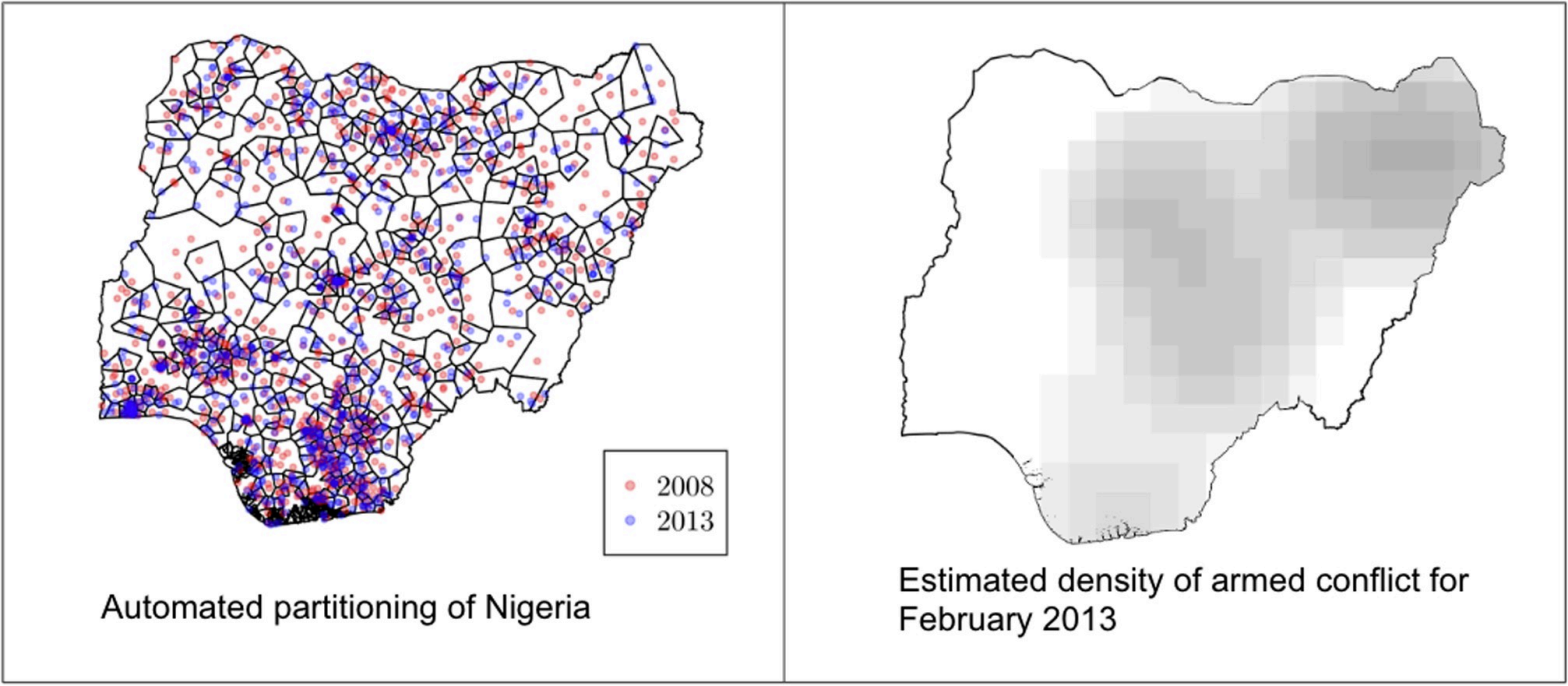
(I) Automated partitioning of Nigeria and (II) estimated density of armed conflict in Nigeria for a particular month. Left: the partitioning algorithm tries to maximize the number of resulting areas under the condition that all areas must include observations from both DHS 2008 and 2013. Right: estimated density of armed conflict. The displayed month is February 2013, and based on an observational window of 12 months.

For each month and area of Nigeria we calculate the conflict intensity for each observation (i.e. child) in our data. To derive our armed conflict intensity variable we treat actual battles and killings as manifestations of a latent point process, and we estimate the density of this point process for all months of which we have DHS observations in Nigeria. The density of the point process can be utilized to predict the number of events (i.e. persons killed) in a given cell of a grid for a given time span, and we ascribe a conflict intensity value for each child by linking the location of the child to that grid. The time span that we use is 12 months preceding the DHS interview as armed conflict causes long term damage of infrastructure, institutional weakness, and devastates vegetation, land, water and thereby agriculture, making it difficult to return to normalcy once the conflict subsides. Since the date of the DHS interview varied for each cluster—moreover, that the observational period spanned from June to November 2008 and from February to July 2013—we collected the 12 months preceding each interview to derive the values for conflict intensity. Fig 1 shows the partitioning we use and the estimated intensity of armed conflict for one of the months in the dataset.

### Flood and drought data

The African Flood and Drought Monitor (AFDM) monitors and predicts meteorological, agricultural and hydrological drought and flood indicators for Africa [17]. Natural disasters such as droughts account for over 80% of the affected population by disasters in sub-Saharan Africa. This is higher than any other disaster type in Africa [25]. Drought is therefore a key covariate. Furthermore, Nigeria had also experienced its worst flooding in more than 40 years, from July to October 2012, resulting in a devastating humanitarian crisis. Estimated 7 million people were affected by the floods and food prices in flooded parts had risen by 30 to 70 percent [26]. Another flood was reported in 2007. Both floods and droughts severely threatens food and water security and can impact nutritional status (e.g. floods affect agricultural land, tools, and seeds).

From the AFDM we collect drought and flood data for Nigeria for the period of our analysis (2008, 2013). Our drought variable is captured with the Drought Index (%), measuring the severity of drought. Our flood indicator involves Surface Runoff (mm/day), which captures excess water from, for instance rain, that does not infiltrate into the soil due to saturation or high intensity but instead flows overland. For the variables flood and drought we obtain the data for each month were we apply, respectively, the max and min (since this is how they are constructed) of the one year window (retrospective) to capture the longterm effect of flood and drought.

### Conditional average treatment effect

A treatment effect is the average causal effect of a variable on an outcome variable of interest (e.g. an experimental drug for the treatment of an illness). For example, in clinical trials, differences in patient characteristics can effectuate heterogeneous responses to the treatment, of which many factors cannot be directly examined and tested (that is, they are omitted). In the absence of a randomized control trial or a natural experiment (e.g. instrumental variables, regression discontinuity design, difference-in-difference), scholars have to rely on other techniques to model treatment effects.

**Fig 2:**
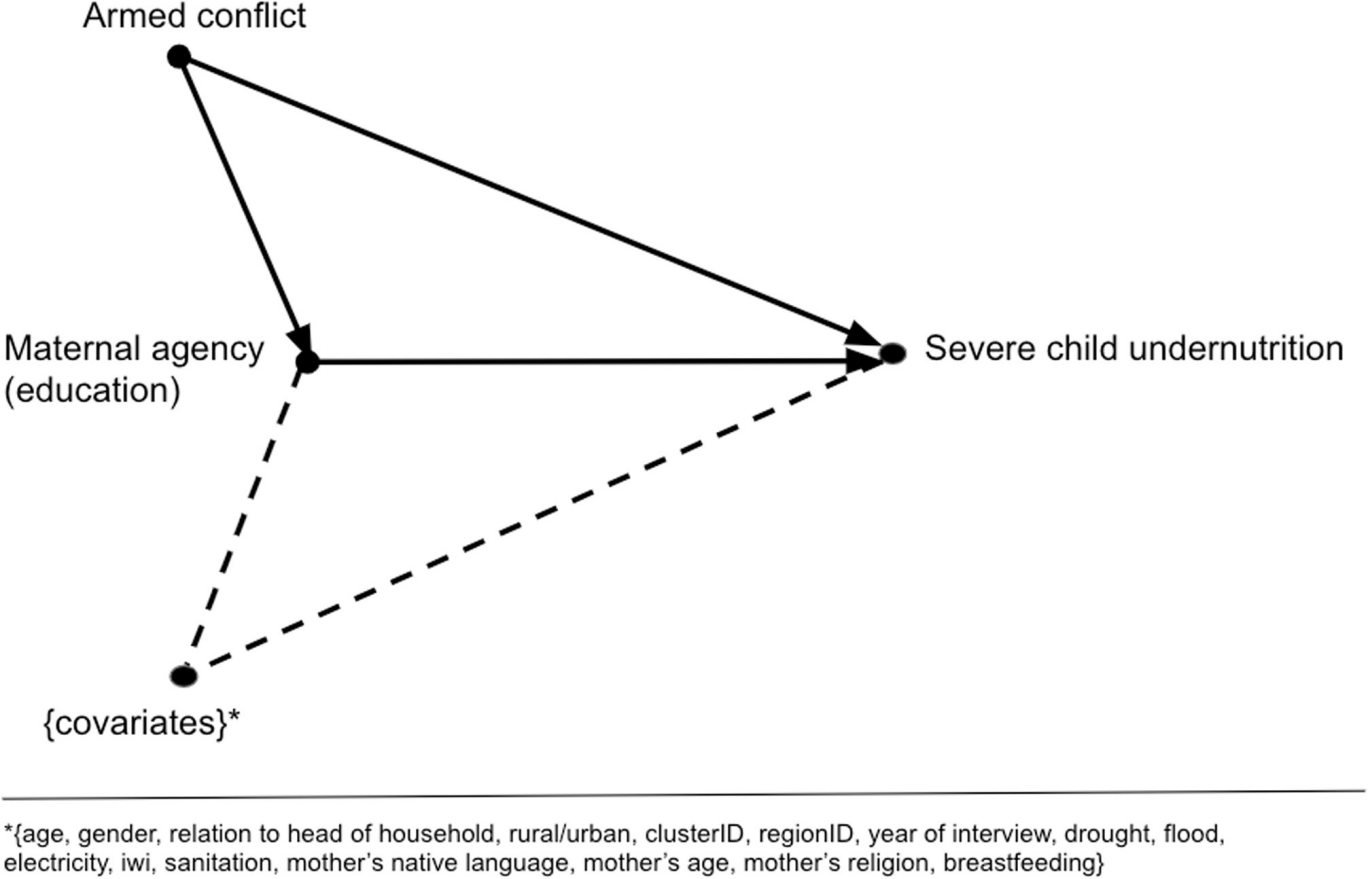
Directed acyclic graph (DAG) on the effect of maternal agency (education) on severe child undernutrition.

For observational studies, omitted variable bias can be identified with the help of theory. Omitted variables, or confounding variables, are factors that affect the treatment and the outcome simultaneously. Recall that the interest of this study is to explore treatment effect heterogeneity, such that we estimate the conditional average treatment effect (CATE). An established way to articulate causal relationships explicitly are directed acyclic graphs (DAGs) [27]. Our research design identifies a treatment effect if, and only if, our proposed DAG holds in reality and this is an untestable assumption, see Fig 2.

Our treatment is causal, to the extent that our assumptions as specified in the DAG fulfills *ignorability* [28]. We use the potential outcomes framework to conceptualize causality. Let an individual with certain characteristics be denoted by *i*. Let D be the treatment at two levels {0,1}; with treatment (D = 1) or no treatment (no education, D = 0) and *x* be the covariates we condition on. Let *d* denoting the observed value; in essence this represents the educational level ranging from 0 years education (control) to more than 12 years education (cut-offs can be retrieved from the section ‘Child, maternal, and household data’). Let the potential outcomes corresponding to the treatment be denoted as {Y_i_(1), Y_i_(0)}. The potential outcome of this study is binary, such that E[Y_i_(D)|X] is the probability Pr(Y_i_ = 1|D = d, X = x)) from a child having an educated mother. The function as specified by g(), see below, is actually a probit that we will convert later into a probability. Running the experiment once, would yield an individual treatment effect, $\theta_{\text{i}}={\text{g}}_{1}({\hat{\text{Y}}}_{\text{i}}\left(1\right)|{\text{X}}_{\text{i}}=\text{x})\;-{\text{g}}_{0}({\hat{\text{Y}}}_{\text{i}}\left(0\right)|{\text{X}}_{\text{i}}=\text{x})$. Running the experiments many times, in large finite samples, we aim to capture the expectation over many individuals, $g_{1}\left(E\left[{\hat{Y}}_{i}\left(1\right)|X_{i}=x\right)\right]-g_{0}\left(E\left[{\hat{Y}}_{i}\left(0\right)|X_{i}=x\right)\right]$.

The outcome variable in this study represents severe child undernutrition, and as described previously, maternal agency (i.e. education) tends to positively influence a child’s nutritional status. The treatment effect can, however, differ within groups that share certain characteristics. For instance, previous work demonstrated that for different levels of education different outcomes in child nutritional status can be observed [10]. In addition, another study on armed conflict and child health showed that children born during the war and living in a war region had 0.42 standard deviations lower height-for-age z-scores compared to those born in a non-war region in the same country [29]. Generally, in treatment effects, accounting for modification may be required even when the variable is not a risk factor for severe child undernutrition. In this application we investigate the treatment effect under the exposure of armed conflict.

### Bayesian additive regression trees

The machine learning literature proposes Bayesian Additive Regression Trees (BART) as a promising avenue to identify hetereogenous treatment effects [30, 31]. The work of Hill [31] provides elaborate description and demonstration of the method with comparison to linear regression (with interaction terms), propensity score matching and learning techniques such as random forests, boosting, neural networks, and support vector machines [30]. Some key characteristics of BART is that it does not require parametric assumptions, which can reduce the risk of model misspecification, and it can capture effects non-parametrically. S2 Appendix can be retrieved for supporting information on tree structures.

In the current case of this study, we model a binary outcome version of BART for classification, with severe child undernutrition constituting {Y(1), Y(0)}. For this binary classification problem we assume a probit model (subscript *i* removed for simplification):

$$\begin{array}{c} p\left(X,D\right)\equiv P\left[\hat{Y}=1|D=d,X=x\right]=\theta [\sum_{j=1}^{m}g\left(d,x;T_{j},M_{j}\right)] \end{array}$$

we split the joint distribution into treatment and control, such that

$$\{\begin{array}{c} \hat{Y_{1}}=g_{1}\left(x;T,M\right)\\ \hat{Y_{0}}=g_{0}\left(x;T,M\right) \end{array}$$

with *θ*[.] is the standard normal cumulative distribution function. Let *T* denote a tree consisting of internal nodes, leaf nodes, and the decision rules that connect the nodes. Let *M* be the fitted values for the *b* terminal nodes of *T*. D denotes the treatment (i.e. treatment or control), and *X* are the values we condition on. When an observation—and in our case this is child with certain characteristics *x* and treatment *d*—goes through the model until it gets placed in a leaf node. Once it reaches the leaf node it reports the *u*_z_ of that leaf node, which is represented in the output function g(d, x; T, M). Since BART fits multiple trees (with 200 as default) the above equation is written as the sum of *m* trees; $\sum_{\text{j}=1}^{\text{m}}$. The default parameters produce efficient performance and do not require cross-validation [30].

When we run BART, we give it our *N* x *K* matrix of covariates that also include the treatment variable, and a vector of size *N* (= observations) that holds the outcome *Y* severe child undernutrition {0,1}. To estimate the conditional average treatment effect (CATE) we follow the procedure similar to that of Hill [31], who describes a simple modeling approach to estimate CATE with BART. More specifically, we give BART a matrix containing the explanatory variables for both the treated and untreated children as training set, and their outcome *Y* in a separate vector. The test includes the explanatory variables of a sample of children of which we store both their original copy and their counterfactual (i.e. two identical matrices bound together). The counterfactual we construct similar to Hill, such that we set the treatment to zero or to another treatment(-level), depending on the analysis. The other variables remain unchanged, at their observed levels.

Of each BART model, we obtain after a burn-in phase of 1,000 iterations, from the posterior distribution, the posterior draws (*N* = 1,000) of the predicted value for each child and its counterfactual. The predicted values are in probit, and to obtain a probability we apply the normal cumulative distribution function (pnorm) to the values. Next, we apply the average over the observations and their counterfactuals, separately. After obtaining for each child the average of its observation and its counterfactual we substract them to obtain the difference, and let this be the individual treatment effect we are interested in. To obtain the CATE the average is applied over the individual effects and we report this with the credible region, denoting the degree of certainty around the probability.

**Fig 3:**
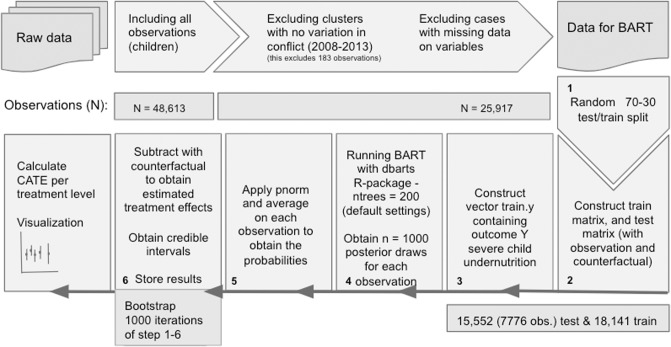
Pipeline overview of data processing and analysis.

Notably, in our procedure, the train and test sampling procedure differs with that of Hill, such that we do not test on a subset of the same sample on which we train, but instead randomly sample 70 percent of the data to the training and 30 percent to test. The testing will therefore be carried out ‘out-of-sample’. In total we obtain a sample of 25,917 observations of children under five in Nigeria. After the randomized train and test split we obtain 7776 observations included for testing (including counterfactuals this involves 15,552 in total) and 18,141 for training. In addition, we apply bootstrapping with 1,000 iterations of the BART model. The bootstrapping results in about 7.8 million observations being processed and for each, from the posterior draws, the average probabilities are calculated. We apply these procedures to increase generalization of the results. For an illustrative overview of our pipeline see Fig 3. Our analysis is carried out in R version 3.5.1 [32] and R package dbarts version 0.9-5 [33].

**Fig 4:**
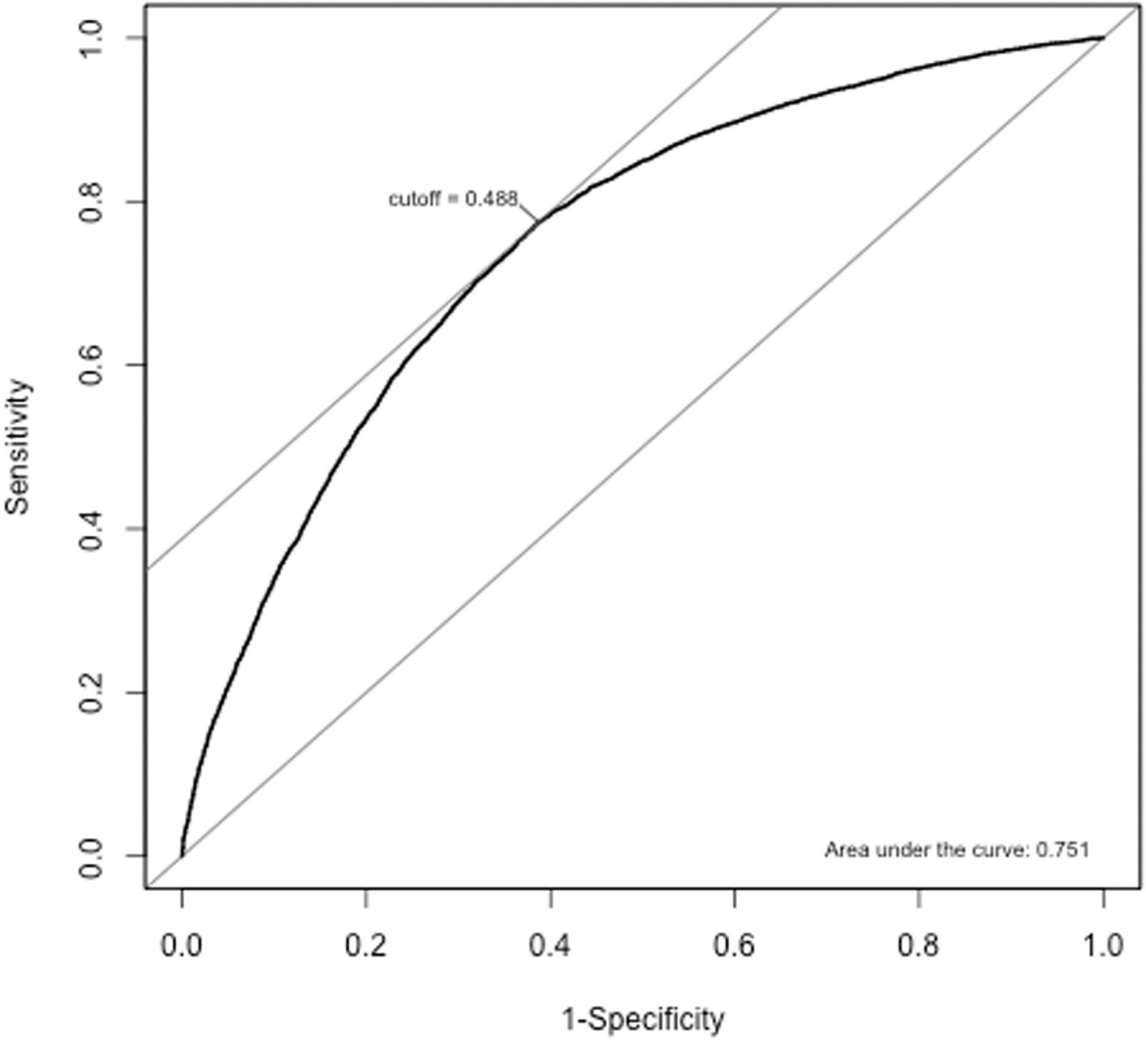
Area under the ROC curve sensitivity = 77.5%, specificity = 61.3%, AUC = 0.751 with cutoff = 0.488.

To check the model’s performance on predicting the outcome we use the measures sensitivity and specificity and plot the area under the ROC curve. The results estimate the sensitivity and specificity to be 77.5% and 61.3%, respectively. The area under the ROC curve can be obtained from Fig 4.

## Results

**Fig 5:**
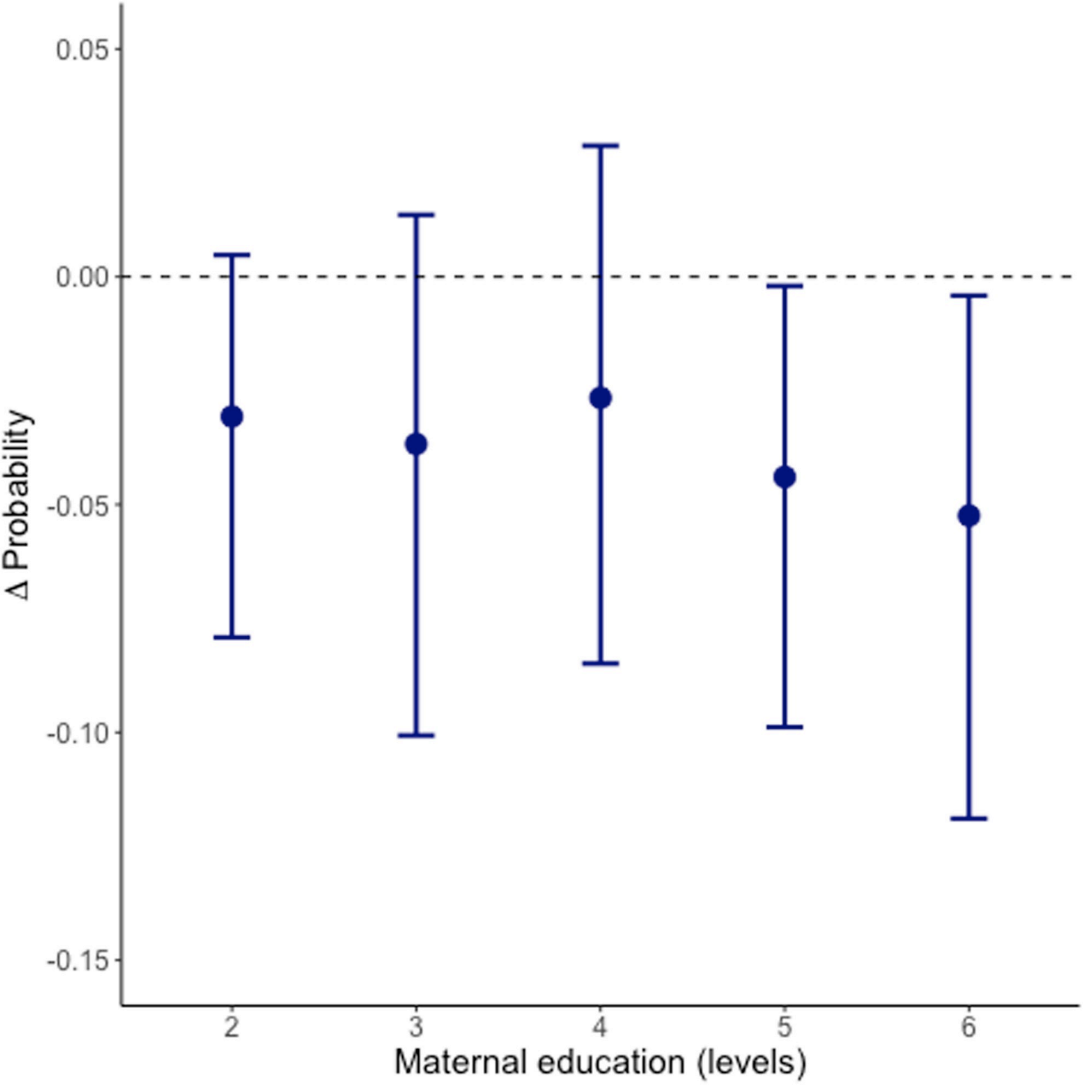
Effect-size (Δ Probability) of maternal education on the treated. A negative Δ probability values implies a beneficial effect: children are less likely to be severely undernourished. The educational levels 2-6 represent the treated, i.e. those with an observed non-zero level of education (retrieve from Table 1 the education level thresholds). In text we provide the results for untreated children (educational level 1), which had a different treatment and control set-up.

The treatment effects of maternal education on the treated children can be obtained from Fig 5. For the untreated children, those who have mothers with zero years of education, our analysis differed. The observed level was used as control setting, and Nigeria’s educational policy of 9 years was set as the treatment. The effect of this level is estimated to be -0.025 with credible intervals 95% [-0.085 - 0.031]. Table 1 provides effect sizes with credible regions for all *other* educational levels.

**Table 1:** Δ Probability by maternal education level.

Level	Maternal Education Level	Treatment^1^	Δ Probability^2^(2.5%–97.5%)
	**Up to and including primary education**		
2	Observed level: (1-6 years)	1-6 years	-0.031 (CI: -0.079, 0.005)
	**Lower secondary education**		
3	Observed level: (7- <9 years)	7-<9 years	-0.037 (CI: -0.101, 0.014)
	**Lower secondary education completed**		
4	Observed level: (9 years)	9 years	-0.027 (CI: -0.085, 0.029)
	**Up to and including higher secondary education**		
5	Observed level: (10-12 years)	10-12 years	-0.044 (CI:-0.099, -0.002)
	**College and higher**		
6	Observed level: (> 12 years)	>12 years	-0.052 (CI: -0.119, -0.004)

^1^ Control = 0 years of education (for all of the above).

^2^ A negative Δ probability value indicates a positive effect of the treatment on the outcome severe child undernutrition.

The treatment effect of maternal education on severe child undernutrition is negative, albeit small, for all observed groups (Table 1). The treatment magnitude shows that children whose mothers have completed more than 9 years of education (educational levels 5 and 6) have significantly lower probability of undernutrition than the other educational levels. Maternal education of 10 to 12 years reduces the probability of the child facing severe undernutrition by about -4%. For maternal education of more than 12 years, this effect increases to -5%. Respective credible intervals can be obtained from Table 1.

**Fig 6:**
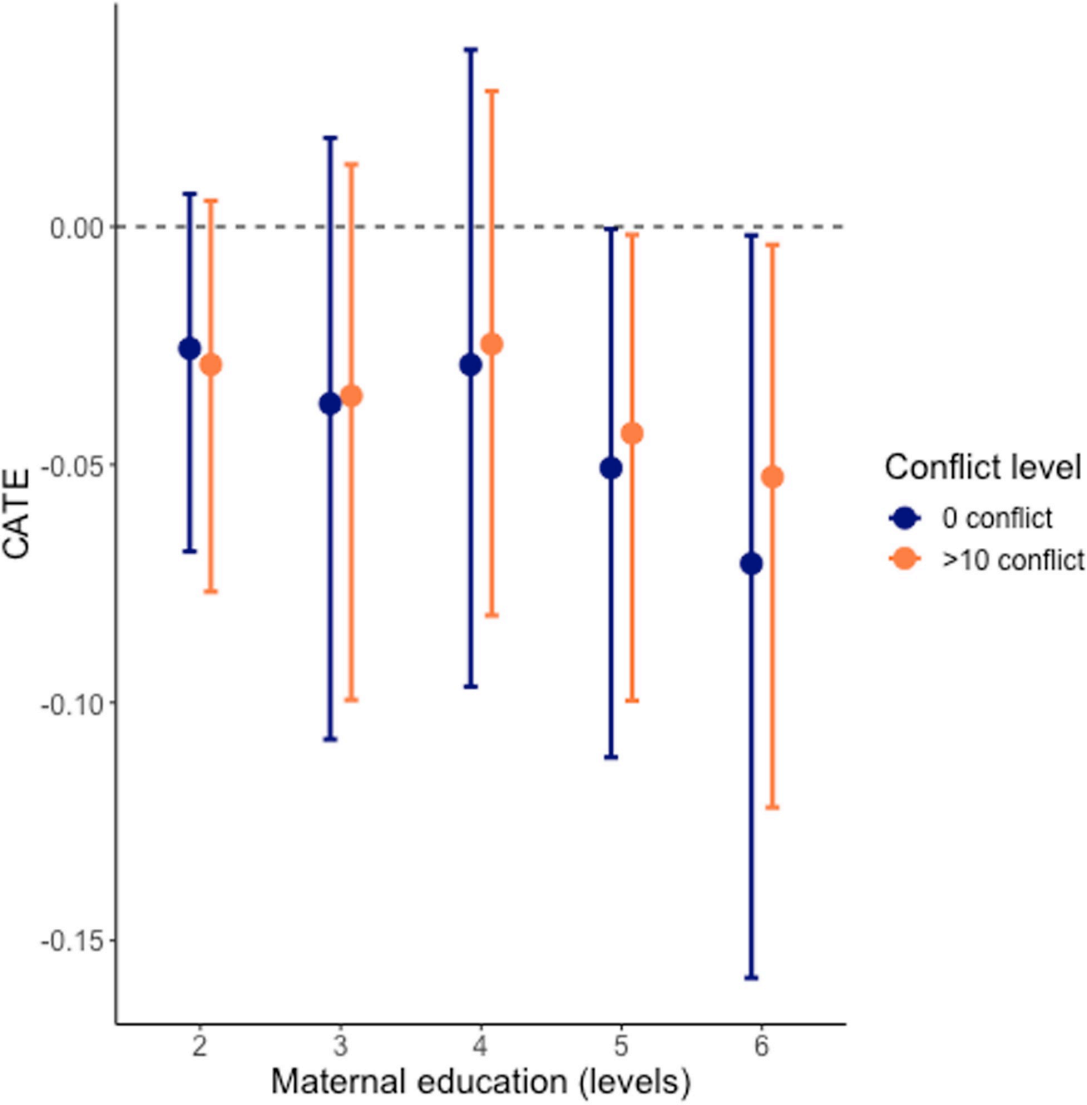
Conditional average treatment effect (CATE) of maternal education under conflict) blue = zero level of conflict, red = more than 10 on the conflict intensity scale.

We are also interested in whether we can observe treatment effect heterogeneity in maternal education for the exposure or absence of armed conflict. Since intensity is a density, we identified the effect inductively, in lack of a theoretical guidance. We plotted the density, and identified that the distribution was skewed to the right. For real-world data this is common, and to include enough observations, yet making the exposure evident, we chose a conflict intensity of 10 to be the minimum for inclusion. Fig 6 shows the effect for those who were having at least some level of education (i.e. zero education excluded) under the exposure of no conflict versus conflict (> 10). This finding highlights that armed conflict does not seem to significantly affect the effect of maternal education on severe child undernutrition.

**Fig 7:**
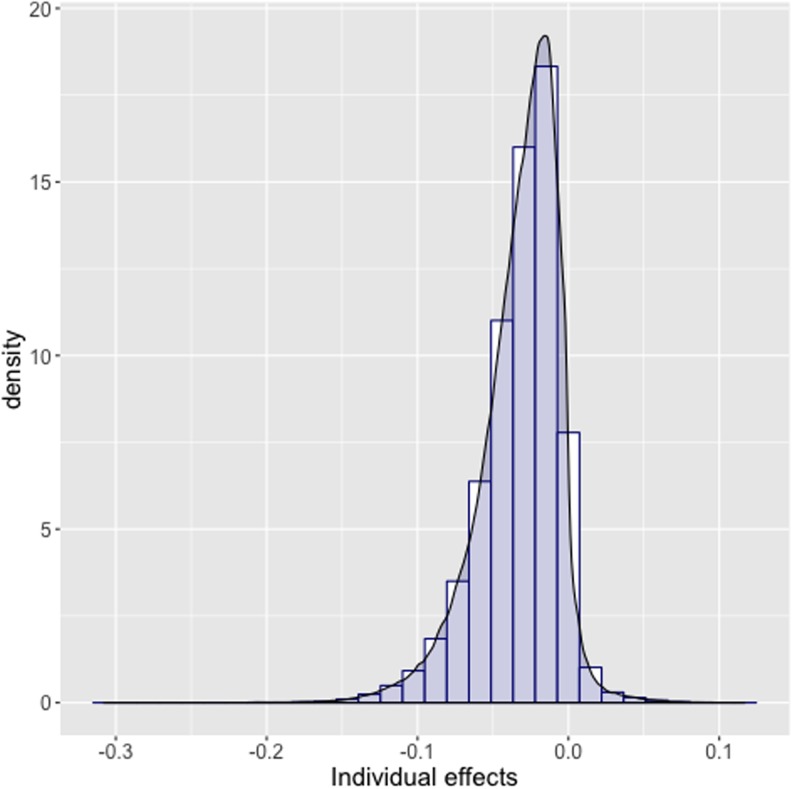
Histogram with density plot of the individual treatment effects.

The previous graph (Fig 6) is helpful to visualize and comprehend the effect for the variable we condition on, however the overall amount of heterogeneity cannot be inferred from this. Fig 7 shows that the estimates do not vary greatly among the observations.

## Discussion

This study analyzed the heterogeneous treatment effect of maternal agency (i.e. education) on severe child undernutrition in Nigeria, and how the effect plays out in a context of armed conflict. We utilized Bayesian Additive Regression Trees (BART) to identify this heterogeneity. We found that maternal education protects children from severe child undernutrition, but only when a mother accumulates more than 9 years of education; that is 10 years of education and higher. Under the exposure of conflict we observed that this beneficial effect diminishes somewhat among children with higher educated mothers, however, this difference is statistically insignificant. Our analysis demonstrates the importance of education both in the absence and presence of conflict.

Previous studies have shown the importance of maternal education but lacked a clear identification of how much education a mother needs to protect her children from the adversity of conflict and turmoil. Our finding identifies such a threshold. It resonates with Makoka and Masibo’s study [10] that found a similar threshold: ten years of education is necessary to prevent stunting. Our study takes the analysis further by identifying heterogeneity within a context of armed conflict. Despite the destructive impact of conflict, mother’s increased education translates to protecting their children [29]. Typically, educated women have less children to feed and look after [34], and at high levels of education, mothers have sound knowledge and practice of feeding and health management of their children [10, 13]. They are also more likely to have a say in household resource management. The aforementioned matters particularly in the presence of external shocks, when governments typically allocate more money for defense and less on social spending [35].

It is important to note at least two limitations of this study. First, we do not have a panel of children and mothers. Individuals included in the 2008 are not the same as in 2013. This precludes direct analysis of the treatment effect of education, before and after a conflict. We model this effect instead by including time as a control, necessitating a DAG that controls for time. Second, the DHS does not directly survey displaced or families living in refuge camps. This means that the effect of conflict can be biased downwards: that is, if we accounted for displaced families the effect of conflict can be larger since households in camps have significantly less agency than households located in their original homes.

A strength of this study is that BART offers a robust solution to model CATE, as additive functions are being discovered by the model rather than specified by the researcher. Finding a model fit (specification searches) in regression strategies may introduce bias as researchers’ assumptions influences the choice of model specification [36], which may in turn potentially produce imprecise effect estimates. Additionally, the use of BART made it possible to include a range of variables we assumed, as outlined in the DAG, to be of importance to fulfill the ignorability assumption and for confounding. Since BART uses a machine learning pipeline with regularizing and out-of sample prediction, it will downgrade variables that are less important in predicting the outcome. With the application of a train-test split and bootstrapping we increased the generalization of our findings. In contrast, ordinary regression studies perform testing in-sample, which can lead to over-fitting and bias [31].

With respect of follow-up research on this topic we encourage replication and improvement when additional repeated surveys become available. As the conflict related to Boko Haram further increased to higher levels after 2013. Such an analysis should also consider the availability and inclusion of data relating to food supply programs in the affected areas. Another valuable contribution would be to deploy a similar design for the entire sub-Saharan Africa. Such a study would test the generalizability of our findings.

Lastly, in terms of policy recommendations, our study shows the importance of shifting the policy debate towards not merely policymakers delivering a one-off education package to their population, but rather building length and quality into this package. We are calling for a shift in nuances about the policy debate. Most policymakers would agree that basic education is key for development. However, our finding is supporting the idea that basic education is not sufficient. Our study shows that protective effect of education sustains even during times of conflict. This effect multiplies if women had access to higher education. They can then start influencing different parts of society: from politics to the economy. This would not only provide them with the tools to build their own agency; it would also be an investment in future generations.

## Supporting information

S1 Appendix Household, mother and child characteristics. (PDF)

S2 Appendix Bayesian Additive Regression Trees (BART): Supporting information on tree models. (PDF)
