# Supplementary material for: The influence of maternal agency on severe child undernutrition in conflict-ridden Nigeria: Modeling heterogeneous treatment effects with machine learning
Source: PLoS One. 2019 Jan 9;14(1):e0208937. doi: 10.1371/journal.pone.0208937 (PMC6326456; doi:10.1371/journal.pone.0208937)
Supplement: S1 Appendix — (PDF) [file pone.0208937.s001.pdf]

S1 Appendix. Household, mother and child characteristics

Table 1. Overview of household, mother and child characteristics (N=25,917)

	N(%) or mean(sd)
**Household size (number of people)**	6.4 (3.2)
**Religion**	
Islam	16790 (64.8%)
Traditional religion	6734 (26.0%)
Catholicism	2384 (9.2%)
Other	11 (<0.1%)
Christianity	0 (0%)
No religion	0 (0%)
**Native Language**	
Hausa	14005 (54.0%)
Yoruba	5558 (21.4%)
Igbo	4945 (19.1%)
Tiv	448 (1.7%)
Kanuri	434 (1.7%)
Ibibio	401 (1.5%)
English	126 (0.5%)
**Region**	
North west	11539 (44.5%)
South west	5066 (19.5%)
South east	4032 (15.6%)
North east	2539 (9.8%)
North central	1880 (7.3%)
South south	861 (3.3%)

Continued on next page

Table 1 – continued from previous page

	N(%) or mean(sd)
**Rural or urban**	
Rural	15768 (60.8%)
Urban	10149 (39.2%)
**Electricity status**	
Has electricity	13989 (54.0%)
Lacks electricity	11928 (46.0%)
**International wealth index**	40.7 (22.5)
**Conflict: intensity (12 months preceding)**	7.8 (10.9)
**Drought: index (%) (12 months preceding, min)**	26.3 (13.2)
**Flood: surface runoff (mm/day) (12 month (...), max)**	1.27 (1.5)
**Mother's age (years)**	29.3 (6.8)
**Mother's education level**	
No education (0 years)	12079 (46.6%)
Up to and including primary education (1-6 years)	5233 (20.2%)
Lower secondary education (7-<9 years)	639 (2.5%)
Completed lower secondary education (9 years)	980 (3.8%)
Up to and including higher secondary education (10-12 years)	5130 (19.8%)
College and higher (more than 12 years)	1866 (7.2%)
**Mother currently breastfeeding**	15118 (58.3%)
**Child's age (years)**	1.9 (1.4)
**Child's gender**	
Male	13037 (50.3%)
Female	12880 (49.7%)
**Child has severe undernutrition**	9520 (36.7%)
**Child has severe sanitation deprivation**	5576 (21.5%)
**Child's relationship to head of household**	
Son/daughter	23,908 (92.2%)
	Continued on next page

Table 1 – continued from previous page

	N(%) or mean(sd)
Grandchild	1609 (6.2%)
Adopted/foster/stepchild	62 (0.2%)
Brother/sister	17 (0.1%)
Niece/nephew	182 (0.7%)
Other relative	93 (0.4%)
Not related	46 (0.2%)
