# Supplementary material for: The influence of maternal agency on severe child undernutrition in conflict-ridden Nigeria: Modeling heterogeneous treatment effects with machine learning
Source: PLoS One. 2019 Jan 9;14(1):e0208937. doi: 10.1371/journal.pone.0208937 (PMC6326456; doi:10.1371/journal.pone.0208937)
Supplement: S2 Appendix — (PDF) [file pone.0208937.s002.pdf]

S2 Appendix. Bayesian Additive Regression Trees (BART): supporting information on tree models

The intuition behind BART is regression and classification tree models [1]. Tree models consist of a structure and decision rules that lead down to the bottom node, and by repeatedly splitting into smaller subgroups it can explain variation in an outcome. The root node is the starting point of the tree and a node is a subset of the data fed to the model. There are two types of nodes: a terminal (leaf) node and a nonterminal (internal) node. The former makes the splits on the basis of a Boolean question about a single predictor, and this can be different for the type of variable (binary, continuous, categorical). The latter can be perceived as the final result of a combination of decisions, where the sample has been split into a more homogeneous subgroup. Another term is 'target variable' which can be framed as the outcome that we model, and in BART this can be both for modeling binary and continuous outcomes. The branches of the tree represent the path, and decision rule, from the root node through internal nodes to a leaf node. The larger a tree grows the more it becomes prone to overfitting, and also small changes of the sample can lead to the grow of a different tree, which both pose limitations on generalization of model results. To make the results less dependent on the sample a tree can be 'pruned', which entails finding an optimum tree. Another strategy is to take the sum of trees, which is what is applied with BART.

[1] Breiman L, Friedman J, Stone CJ, Olshen RA. Classification and Regression Trees. New York: Chapman & Hall; 1984.
